# Epidemiological and clinical aspects of Guillain-Barré syndrome and its variants

**DOI:** 10.1590/0004-282X-ANP-2020-0314

**Published:** 2021-06-23

**Authors:** Dayanne Rodrigues da Cunha Alves Bento Oliveira, Rubens Nelson Morato Fernandez, Talyta Cortez Grippe, Fabiano Silva Baião, Rafael Lourenco Duarte, Diego Jose Fernandez

**Affiliations:** 1 Hospital de Base do Distrito Federal Instituto de Gestão Estratégica em Saúde do Distrito Federal Departamento de Neurofisiologia Clínica Brasília DF Brazil Hospital de Base do Distrito Federal, Instituto de Gestão Estratégica em Saúde do Distrito Federal, Departamento de Neurofisiologia Clínica, Brasília DF, Brazil.; 2 Centro Universitário de Brasília Faculdade de Medicina Brasília DF Brazil Centro Universitário de Brasília, Faculdade de Medicina, Brasília DF, Brazil.; 3 Hospital da Força Aérea Brasília DF Brazil Hospital da Força Aérea de Brasília, Brasília DF, Brazil.; 4 Secretaria Municipal de Saúde de Anápolis Anápolis GO Brazil Secretaria Municipal de Saúde de Anápolis, Anápolis GO, Brazil.; 5 Centro de Diagnóstico por Imagem de Goiânia Goiânia GO Brazil Centro de Diagnóstico por Imagem de Goiânia, Goiânia GO, Brazil.; 6 Instituto de Neurologia de Goiânia Goiânia GO Brazil Instituto de Neurologia de Goiânia, Goiânia GO, Brazil.

**Keywords:** Guillain-Barré Syndrome, Electromyography, Neurophysiology, Epidemiology, Síndrome de Guillain-Barré, Eletromiografia, Neurofisiologia, Epidemiologia

## Abstract

**Background:**

Guillain-Barré syndrome (GBS), an acute polyradiculoneuropathy that occurs because of an abnormal inflammatory response in the peripheral nervous system, is clinically characterized by acute flaccid paresis and areflexia with or without sensory symptoms. This syndrome can lead to disabling or even life-threatening sequelae.

**Objective:**

This study aimed to present the clinical and epidemiological aspects of GBS in patients admitted to a tertiary-level hospital in the Federal District between January 2013 and June 2019.

**Methods:**

In this observational, cross-sectional and retrospective study, medical records of patients diagnosed with acute inflammatory demyelinating polyradiculoneuropathy, acute motor axonal neuropathy or acute axonal motor-sensitive neuropathy based on electromyographic findings were included, and clinical data were collected retrospectively.

**Results:**

A total of 100 patients (63 males and 37 females; ratio, 1.7:1) aged 2–86 years (mean, 36.4 years) were included. The mean annual incidence rate of GBS was 0.54 cases/100,000 inhabitants, with 52 and 49% of the cases occurring between October and March (rainy season) and between April and September (dry season), respectively. The proportions of patients showing each GBS variant were as follows: demyelinating forms, 57%; axonal forms, 39%; and undetermined, 4%. The mean duration of hospitalization was 8–15 days for most patients (38%). During hospitalization, 14% of the patients required mechanical ventilation and 20% experienced infectious complications.

**Conclusion:**

The findings indicate that there was an increase in the incidence of GBS during the rainy season. Moreover, we did not observe the typical bimodal distribution regarding age at onset.

## RESUMO

**Introdução:**

Síndrome de Guillain-Barré (SGB), uma polirradiculoneuropatia aguda que ocorre devido a uma resposta inflamatória anormal no sistema nervoso periférico, é caracterizada clinicamente por paralisia flácida aguda e arreflexia, com ou sem sintomas sensitivos. Essa síndrome pode deixar sequelas incapacitantes ou até ameaçar a vida.

**Objetivo:**

Apresentar os aspectos clínicos e epidemiológicos da SGB em pacientes internados em um hospital terciário do Distrito Federal, no período de janeiro/2013 a junho/2019.

**Métodos:**

Estudo observacional, transversal e retrospectivo, no qual pacientes com diagnóstico de polirradiculoneuropatia desmielinizante inflamatória aguda, neuropatia axonal motora aguda ou neuropatia axonal sensitivo motora aguda a partir dos achados eletroneuromiográficos foram selecionados e seus dados clínicos coletados retrospectivamente em seus prontuários.

**Resultados:**

Um total de 100 pacientes (63 homens e 37 mulheres; proporção de 1,7:1), com idades entre 2–86 anos (média, 36,4 anos), foram incluídos. A taxa média anual de incidência de SGB foi de 0,54 casos/100.000 habitantes, com 52 e 49% dos casos ocorrendo entre outubro e março (período chuvoso) e entre abril e setembro (período seco), respectivamente. A proporção de pacientes que apresentaram cada variante de SGB foi a seguinte: formas desmielinizantes, 57%; formas axonais, 39%; e indeterminado, 4%. A duração média da hospitalização foi de 8‒15 dias para a maioria dos pacientes (38%). Durante a hospitalização, 14% dos pacientes necessitaram de ventilação mecânica e 20% apresentaram complicações infecciosas.

**Conclusão:**

Os resultados indicam aumento na incidência de GBS durante a estação chuvosa. Além disso, não observamos a distribuição bimodal típica em relação à idade de início.

## INTRODUCTION

Guillain-Barré syndrome (GBS) is an acute immune-mediated polyneuropathy characterized by flaccid and rapidly progressive paresis that is symmetrical, ascending and areflexic^1^. In two-thirds of the patients, upper respiratory tract infection (URTI) or diarrheal disease (usually caused by *Campylobacter jejuni*) occurs 1–4 weeks before the onset of neuropathy^2^. Cases of GBS have also been reported soon after administration of the rabies vaccine, as well as with certain vaccines against the influenza A virus. Similarly, possible associations with acute arbovirus infections, including Zika and chikungunya, have been extensively studied in recent years^3^.

GBS is mediated by humoral and cellular responses that directly destroy the myelin sheath of axons of peripheral nerves^4^. Although GBS variants share immunomediated pathogenesis, they differ in their pathophysiology, clinical presentations and endpoints, and are classified into different subtypes. For example, immune reactions against epitopes of Schwann cell surface membranes or myelin result in demyelinating neuropathy, while those directed against axonal membrane antigens cause the acute axonal form of the syndrome^5^.

However, over recent years, microstructural changes in the nodal region have been discussed as the key to understanding the pathophysiology of neuropathies associated with ganglioside antibodies, and a new category of nodo-paranodopathies has been proposed, in order to better characterize these disorders. The concept of nodo-paranodopathies seems appropriate for several acute and chronic neuropathies associated with antibodies against gangliosides and paranodal axo-glial proteins, as this concept focuses on the site of the primary nerve injury while considering the specific pathophysiological mechanisms, reconciling morphological contrasts and electrophysiological findings, and avoiding wrong diagnoses^6^.

In North America and Europe, the demyelinating form, i.e. acute inflammatory demyelinating polyradiculoneuropathy (AIDP), is predominant. The less common axonal forms are found in only 5% of patients and include acute motor axonal neuropathy (AMAN). Patients with axonal forms of GBS reach the nadir of symptoms earlier than those with the demyelinating form, although the recovery rates for the two forms are comparable^7^. Motor-sensory axonal neuropathy (AMSAN) is considered to be the most severe form of the GBS phenotype and typically exhibits rapid progression to tetraplegia^8^. In Asia and Central and South America, axonal forms constitute 30–47% of cases^1^.

Few studies have evaluated the epidemiology of this syndrome in Brazil^9^^,^^10^^,^^11^. The objective of this study was to demonstrate the clinical and epidemiological aspects of GBS in a series of patients admitted to a tertiary-level hospital in the Federal District.

## METHODS

In this observational, cross-sectional and retrospective study, the medical records of patients diagnosed with AIDP, AMAN or AMSAN, based on the findings from electroneuromyography (ENMG) performed between January 2013 and June 2019, in the neurophysiology sector of a tertiary-level hospital in the Federal District, the only public hospital in the Federal District that performs this examination, were included. The study was approved by the local ethics committee under number 29193019.2.0000.8153.

The median, ulnar, superficial and deep fibular, sural and tibial nerves of all four limbs were analyzed. Regarding motor conduction, distal latency, amplitude (baseline to negative peak), conduction speed and duration (first negative deflection to the baseline of the last negative deflection) of the compound muscle action potential, along with minimum F-wave latency, were analyzed. Regarding sensory conduction, amplitude (baseline to negative peak), onset latency and conduction velocity of the sensitive action potential were analyzed.

The ENMG findings and medical records of 100 patients with a clinical history and findings of physical and cerebrospinal fluid examination compatible with GBS were reviewed in accordance with the Asbury and Cornblath criteria^12^. Patients diagnosed with acute demyelinating or axonal polyradiculoneuropathy due to paraneoplastic conditions, systemic diseases, acquired immunodeficiency syndromes or polyneuropathies that were later diagnosed as chronic demyelinating inflammatory polyneuropathy (CIDP) were excluded.

The following variables were analyzed: age, sex, predisposing factors, form of the disease, electrophysiological characteristics, complications, need for ventilatory support, duration of hospitalization, seasonality and mortality.

As the Federal District is located in a tropical climate region where the seasons are poorly defined, the seasonal distribution of GBS incidence was evaluated in two periods: dry (April to September) and rainy (October to March)^13^.

## RESULTS

### Epidemiological data

Data on epidemiological characteristics, the form of the disease, predisposing factors and duration of hospitalization are presented in Table 1. Among the 100 patients included in this study (mean age, 36.4 years; range, 2–86 years), the number of male patients was predominantly high (male-to-female ratio, 1.7:1.0). The patients were subdivided into four age groups. In the <14 years group, the prevalence of GBS was similar between the sexes; however, in the remaining groups, the number of male patients was predominantly high.

**Table 1:** Clinical characteristics.

Clinical characteristics		(n=100)
Demographic characteristics		%
Age		
	0–14 years	12
	15–34 years	35
	35–59 years	43
	Over 60 years	10
Sex		
	Male	63
	Female	37
Form of disease (%)		
	AIDP	57
	AMAN	24
	AMSAN	15
	Undefined	4
Previous factors (%)		
	URTI	23
	AGEC	26
	Arboviruses	11
	Other (vaccination and pregnancy)	4
	Unknown	36
Duration of hospitalization		
	0 days	2
	1–7 days	27
	8–15 days	38
	16–30 days	23
	≥30 days	10

AIDP: acute demyelinating inflammatory polyneuropathy; AMAN: motor axonal neuropathy; AMSAN: motor-sensory axonal neuropathy; URTI: upper respiratory tract infections; AGEC: acute gastroenterocolitis

The most frequently observed variant of the disease was the demyelinating form, followed by axonal neuropathy (AMAN and AMSAN). Definition of the pathophysiological mechanism underlying 4% of the cases was not possible because ENMG was performed at an early stage. Attempts made to contact these patients for further examination were unsuccessful.

The annual case frequency was 8–23 cases/year; the highest incidence rate was observed in 2013 (23 cases), followed by 2018 (18 cases). The incidence rates remained constant between 2014 and 2016 (13 cases/year) and decreased in 2017 (8 cases). In the first half of 2019, 12 cases were recorded. The average annual frequency of occurrence of GBS was 14.28 cases/year. The average annual incidence rate was 0.54 cases/100,000 inhabitants in the Federal District.

**Figure 1:**
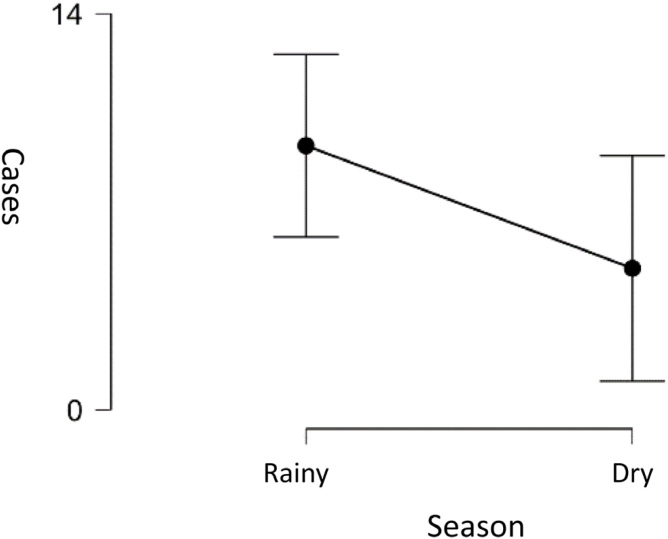
Incidence rate of Guillain-Barré syndrome.

Evaluation of the GBS frequency distribution between the seasons using Student's t-test revealed a significant difference in GBS distribution according to the season (p=0.05; Figure 1). Thus, the season was a relevant factor for case distribution. Student's t-test was chosen because the sample distribution was normal.

**Figure 2:**
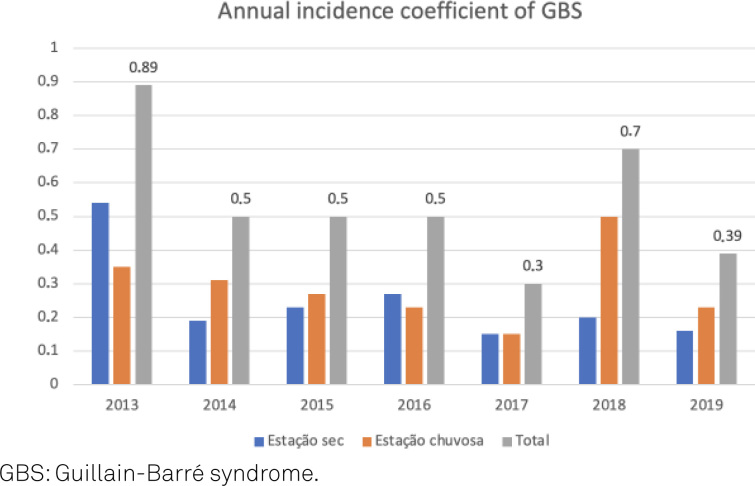
Annual Guillain-Barré syndromeincidence coefficient and Guillain*-*Barré syndromeincidence in the rainy and dry periods from 2013 to 2019 (June).

The mean frequencies of occurrence of GBS were 5±3.98 and 9.3±3.23 in the dry and rainy seasons, respectively. During both these seasons, the demyelinating forms were observed predominantly (50% in the rainy season and 64.5% in the dry season). With regard to the annual incidence rate of GBS, a larger proportion of GBS cases were observed during the rainy seasons of all years except 2013 and 2016; during these two years, 52.1 and 53.8% of the cases, respectively, occurred in the dry season (Figure 2).

#### Predisposing factors

Among the predisposing factors for GBS identified in the 30-day period before the onset of neurological symptoms, histories of upper respiratory tract infections (URTI) and acute gastroenterocolitis (AGEC) showed the highest frequencies. Patients with a history of arbovirus infections (13%) were also observed, with 18 and 19% showing symptoms suggestive of Zika and chikungunya, respectively, and 63% showing serological positivity for dengue fever.

We registered two GBS cases of women with ongoing pregnancies; one was diagnosed with AIDP and the other with AMAN. Additionally, we registered one case of AIDP relating to a woman in the late puerperium. A history of influenza vaccination in the 60 days before the onset of neurological symptoms was identified in one patient. Higher incidence rates of URTI (59%), AGEC (73%), and arbovirus infections (55%) were observed in the rainy season.

#### Complications

Fourteen percent of all the patients required mechanical ventilation at some point during the course of the disease. Among them, 64.3 and 35.7% had demyelinating and axonal forms of the disease. About 20% of all the patients presented with infectious complications during hospitalization, in which the lungs were the most prevalent focus of infection (85%). Among the 57 patients with a demyelinating form of the disease, 24.5% recovered from this complication, compared with 12.8% of the 39 patients with an axonal form.

#### Treatment

Among the 100 patients evaluated, 98 received hospital treatment. The treatment of choice was immunoglobulin-based in 88 patients; however, six patients had to undergo more than one cycle of treatment because of the refractoriness of the condition. None of the patients had complications relating to infusion of the drug. In addition, eight patients underwent plasmapheresis, and only one of them presented with hemodynamic instability during the treatment. In that case, the treatment had to be suspended. No specific treatment (immunoglobulin-based or plasmapheresis) was indicated for two patients because they showed mild clinical symptoms and good recovery.

#### Duration of hospitalization time and form of the disease

As detailed in Table 1, the duration of hospitalization was 8–15 days for most patients. Among those hospitalized for more than 31 days, 70% suffered respiratory failure and consequently required mechanical ventilation during the initial period of hospitalization. In these patients, respiratory failure progressed to infectious complications. Table 2 shows the relationship between the duration of hospitalization and the form of the disease: the duration of hospitalization was higher for axonal forms (22 days) than for demyelinating forms (14 days).

**Table 2:** Duration of hospitalization and form of the disease.

Hospitalization period	Form of disease		
	Demyelinating (n=55)	Axonal (n=39)	Undefined (n=4)
1–7 days	19 (34.5%)	8 (20.5%)	0
8–15 days	19 (34.5%)	16 (41%)	3 (75%)
16–30 days	12 (22%)	10 (25.6%)	1 (25%)
>30 days	5 (9%)	5 (12.9%)	0

### Mortality

In our data, no mortality was observed during the follow-up period (2013–2019).

### Time between symptom onset and electroneuromyography recording

Most patients (n=38) underwent ENMG 8–15 days after the onset of symptoms. Furthermore, 27, 24 and 6 patients underwent the examination 16–30 days, 7 days, and 30–60 days after symptom onset, respectively. While most patients underwent ENMG once, 22 patients underwent a second ENMG. Among these 22 patients, 12 patients underwent the second exam within 60 days; in 41% of the patients in this subgroup, definition of the form of the disease was possible only after performing the second examination.

## DISCUSSION

Previously, a meta-analysis estimated the overall annual incidence rate of GBS to be 0.8–2 cases/100,000 individuals^14^. In Latin America, high incidence rates have been reported in Chile (2.12 cases/100,000)^15^ and Argentina (2.06 cases/100,000)^16^, while a relatively low annual incidence rate has been reported in Brazil (0.4 cases/100,000)^9^. The estimated annual average incidence rate in the Federal District is 0.5 cases/100,000, which is similar to that of Brazil.

The incidence rates differed over the years, with peaks observed in 2013 (0.89 cases/100,000) and 2018 (0.46 cases/100,000) (Figure 1). Most patients with acute paraparesis are evaluated in our hospital. However, there might have been a few evaluated in private hospitals, and the data of these patients were not included while calculating the incidence rates.

The proportions of AIDP and axonal forms of GBS may vary in different countries depending on the climatic conditions, basic sanitation and geographical region of origin of the patient. In North America and Europe, demyelinating forms are predominant (69–90%), while axonal forms are more common in Asia and South America^17^.

In Brazil, a study on GBS conducted in Rio Grande do Norte between 1994 and 2007 showed that demyelinating forms predominated (81.8%) and that motor axonal (AMAN, 14.7%) and motor-sensitive forms (AMSAN, 3.3%) were present^11^. Another study revealed that the highest incidence rate was shown by demyelinating forms (57%), followed by AMAN (24%) and AMSA (15%), in the Federal District^13^. A previous study^14^ reported that men are more susceptible to GBS than women; this finding is in line with that of the current study (male-to-female ratio, 1.7:1).

According to the International Study on GBS^17^, the disease is prevalent in all age categories. The incidence rate increases with age, from 0.6 cases/100,000 people/year for individuals aged <2 years to 2.7 cases/100,000 people/year for those aged >80 years^14^, and shows bimodal distribution with peaks for young (15–34 years) and older (>60 years) adults^18^. On the contrary, we found a higher incidence rate (43%) among individuals aged 35–59 years than among those aged >60 years (10%).

Previous studies have failed to identify a clear relationship between the incidence of GBS and seasons, and such a relationship is variable across countries^17^. However, the association between the occurrence of UPTI before GBS and winter is clear^9^.

Overall, we found that the incidence rate of GBS was higher in the rainy season (52%) than in the dry season (48%). Demyelinating forms were found to be predominant in the dry period (64.5%) while demyelinating and axonal forms showed similar incidence rates (50%) in the rainy season. Although in most years the incidence rates of GBS were higher in the rainy season than in the dry season, the opposite was true in 2013 and 2016 (dry season, 52.1 and 53.8%, respectively). In 2017, the two seasons showed similar rates of GBS incidence (Figure 1).

Other studies have also reported seasonal distribution of incidence rates of GBS. This variability is thought to reflect the variability of predisposing factors, such as infections of the gastrointestinal and respiratory tracts. In our study, this possibility was corroborated by high rates of infections, as predisposing factors during the rainy season^19^^,^^20^^,^^21^^,^^22^^,^^23^^,^^24^.

GBS is typically a post-infection disease characterized by rapid monophasic progression of the disease after infection (interval of <1 month), usually without relapse. Identification of the predisposing factor is important, as it could be correlated with the clinical phenotype and prognosis. The agent most commonly associated with GBS is *Campylobacter jejuni* (specifically serotype O: 19), which causes axonal injury and Wallerian degeneration and is characterized by the presence of GM1 and GD1 antiganglioside antibodies, with a poor prognosis^25^. In the present study, similar observations were made, i.e. 65.3% of cases with a recent history of AGEC progressed to an axonal form of the disease.

Reports on associations between GBS and infections such as dengue, chikungunya, and Zika viruses are now available^26^^,^^27^. In our series of cases, there was a clinical suspicion of involvement of arboviruses in 11% of cases, with predominance in the dry season (63.6%). Among the corresponding patients, 63% tested serologically positive for dengue virus infection, 18% presented with symptoms suggestive of Zika, and 19% were suspected of chikungunya; however, no specific tests were performed for confirmation.

Recently, a nationwide increase in the incidence of GBS following the Zika virus epidemic in 2015 was reported^28^. However, this was not supported by maintenance of annual incidence during this period, in our study (Figure 2).

Reports on the incidence of GBS associated with pregnancy, surgery or trauma are rare^18^. We identified two cases in pregnant women and one case in a woman in the late puerperium; however, no other predisposing factors were identified.

Incidence of GBS shortly after administration of rabies vaccine and various types of influenza vaccines has also been reported. One study reported that the incidence of GBS increased by 1.6 cases/1,000,000 people vaccinated against influenza A virus subtype H1N1 and other seasonal influenza viruses^3^. On the other hand, a recent retrospective evaluation of 3,523 cases of GBS in France showed that there was no association between seasonal influenza vaccination and GBS^29^. In our study, only one patient with a history of influenza vaccination within 60 days before the onset of GBS symptoms was noted; this individual had a demyelinating form of the disease.

GBS is a potentially fatal disease that requires intensive medical care, monitoring of vital signs, management of autonomic dysfunctions and administration of immune treatment. Several randomized clinical trials have evaluated the effects of immunotherapy on GBS over recent decades, and intravenous immunoglobulin therapy and plasmapheresis have been proven to be equally effective^30^. In our study, 98% of the patients were hospitalized, and 89.7 and 8.1% of them underwent immunoglobulin therapy and plasmapheresis, respectively. The medical records did not indicate the treatments that the remaining patients underwent.

Respiratory failure is the most serious short-term complication of GBS. About 20–30% of patients may need invasive mechanical ventilation, which increases the risk of development of complications. Among intubated patients, 60% develop complications such as pneumonia, sepsis, gastrointestinal bleeding and pulmonary embolism^31^. The longer the progression time of AIDP is, the stronger the signs of severity and the indication for intensive monitoring will be^25^. The progression pattern and speed differ between AMAN and AIDP. AMAN shows faster progression and an earlier peak. The current study presented similar findings (requirement for mechanical ventilation, 14%; development of infectious complications, 20%; and pulmonary focus of infection, 85%).

The recovery patterns of patients with AMAN also differ from those with AIDP^32^. The recovery speed of patients with AIDP is relatively uniform, while two patterns of recovery have been observed in patients with AMAN: recovery within a few days and slow and partial recovery. Rapid recovery is caused by resolution of the driving block, and poor recovery is associated with extensive axonal degeneration of nerve roots^33^.

In our study, the number of patients with prolonged duration of hospitalization (>30 days) was similar to that observed in previous studies (axonal forms, 12%; and demyelinating form, 9%). A high proportion of the patients with demyelinating forms of the disease had infectious complications and required mechanical ventilation.

ENMG plays a key role in the evaluation of patients with GBS. The criteria defined for AMAN and AMSAN by Ho and Hadden were based on the initial assumption that these subtypes were pathologically characterized by simple axonal degeneration. However, some patients with AMAN show transient conduction block or deceleration in the intermediate and distal nerve segments, which mimics demyelination but without the development of abnormal temporal dispersion and is referred to as reversible conduction failure (RCF), at the onset of the condition^34^.

The lack of distinction between RCF and demyelinating conduction block leads to erroneous classification of AMAN and RCF as AIDP^34^. Therefore, serial electrophysiological studies are mandatory for diagnosing GBS subtypes, identifying pathophysiological mechanisms and making the prognosis. In our study, defining the disease type of five patients was possible only after performing the second ENMG, while this was not possible in the cases of another four patients because of the lack of a control examination.

Moreover, although we tried to eliminate all the non-GBS polyneuropathies through the ENMG-based inclusion criteria, these criteria carry a lack of sensitivity. Thus, some cases of GBS might be excluded because not all of them fulfil the criteria in all evaluations. It is also important to acknowledge that we did not divide the cases according to the clinical variants because there was a registration bias in some of the medical reports (undisclosed information).

In conclusion, we have presented updated data on the epidemiology, seasonality and electrophysiology of GBS from a representative region of our country. We did not observe the typical bimodal distribution regarding the age at onset. There is a possible association between the incidence of GBS and the rainy season.
